# Improving Molecular Design with Direct Inverse Analysis of QSAR/QSPR Model

**DOI:** 10.1002/minf.202400227

**Published:** 2025-01-11

**Authors:** Yuto Shino, Hiromasa Kaneko

**Affiliations:** ^1^ Department of Applied Chemistry School of Science and Technology Meiji University 1-1-1 Higashi-Mita Tama-ku, Kawasaki Kanagawa 214-8571 Japan

**Keywords:** autoencoder, cheminformatics, drug design, machine learning, virtual screening

## Abstract

Recent advances in machine learning have significantly impacted molecular design, notably the molecular generation method combining the chemical variational autoencoder (VAE) with Gaussian mixture regression (GMR). In this method, a mathematical model is constructed with X as the latent variable of the molecule and Y as the target properties and activities. Through direct inverse analysis of this model, it is possible to generate molecules with the desired target properties. However, this approach outputs many strings that do not follow the simplified molecular input line entry system grammar and generates unrealistic chemical structures in which the properties and activity do not satisfy the target values. In this study, we focus on hierarchical VAE using molecular graphs to address these issues. We confirm that the combination of hierarchical VAE and GMR does not generate invalid outputs and returns molecules that simultaneously satisfy multiple target values. Moreover, we use this method to identify several molecules that are predicted to exhibit activity against drug targets.

## Graphical abstract

We propose a molecular design method that combines hierarchical VAE with GMR, enabling the generation of valid chemical structures that satisfy multiple target properties. This method successfully identifies several molecules with predicted activity against drug targets.

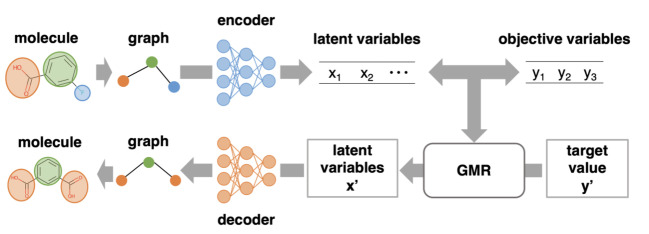

## 1 Introduction

Drug discovery is a complex process involving the design, synthesis, and evaluation of compounds to assess their properties and activity. It has been estimated that the number of theoretically feasible compounds is enormous, ranging between 10^23^ and 10^60^, while the number of compounds to have been synthesized thus far is less than 10^9^ [1]. Therefore, identifying effective drugs in this huge chemical space requires significant amounts of time and money. Additionally, because molecular design is based on the knowledge and experience of researchers, it is difficult to design compounds beyond our current understanding.

Methods for exploring the chemical space efficiently are vital in the field of drug discovery. Consequently, molecular design using machine learning has gained considerable attention. In particular, deep learning‐based generative models have made rapid advances in recent years, significantly enhancing the efficiency with which molecules can be explored for drug discovery and materials design [2].

Various molecular representations are used in generative models. One of the most common is the simplified molecular input line entry system (SMILES), which represents molecules as strings [3]. Another string‐based representation is self‐referencing embedded strings (SELFIES) [4]. Molecules can also be represented as graphs, in which the atoms are nodes and the bonds are edges. An example of a generative model using these diverse molecular representations is the variational autoencoder (VAE). This model learns features from the training data and generates data similar to the training data. A VAE consists of an encoder, which learns to map the input data into continuous latent variables, followed by a decoder, which learns to reconstruct a molecule from its learned latent variables [5]. The chemical VAE (ChemVAE) [6] model has been applied to SMILES generation. Several enhanced VAEs have been developed, including grammar VAE [7],which incorporates grammar rules, and penalized VAE [8], which adjusts the output weights based on the frequency of characters in SMILES. However, these SMILES‐based VAE approaches still struggle to train smooth latent spaces due to the complexity of the grammar involved in SMILES. VAEs can also be employed for graph generation, such as in graph VAE [9], which uses molecular graphs, and JT‐VAE [10], representing substructures as trees with nodes. While these graph‐based approaches have proven effective for the design of small molecules, there exist significant limitations when applied to large and structurally complex molecules. Thus, hierarchical approaches such as hierarchical VAE [11] were developed. Hierarchical VAE incorporated structural motifs, enabling the effective processing of large molecules. Additionally, the NP‐VAE [12] specialized in generating large molecules, such as natural products, by accounting for critical three‐dimensional features such as chirality. MRGVAE [13] incorporated a computational workflow called CReM (chemically reasonable mutations) [14], which enhanced the diversity of generated molecules by introducing local structural mutations. DrugHIVE [15] represented molecules in a grid‐based format, capturing spatial information while considering interactions with proteins, thereby facilitating drug designs. Another generative model used in molecular design is the generative adversarial network (GAN). Compared to VAE‐based methods with loss functions, GANs have a more realistic comparison method to implement adversarial training, which is more interpretable [2]. This model comprises a generator that generates molecules similar to those in the training data and a discriminator that identifies whether these molecules are actually from the training data or have been generated by the generator. The generator is trained to produce increasingly realistic molecules to deceive the discriminator, while the discriminator is trained to identify the difference. Through this competitive learning process, GANs generate realistic molecules that closely resemble the training data. Such molecular generation models have been applied to SMILES [16] and molecular graphs [17]. However, it is difficult for GANs to generate sequences and graphs, compared to VAEs because it requires the gradient to be backpropagated through discrete choices [1, 2]. This is particularly problematic in the generation of molecules, which have a discrete nature.

While significant progress has been made in generating diverse and novel molecules from various molecular representations, the design of molecules with specific properties or activities, known as inverse design, remains equally important. A typical approach to optimizing such properties or activities starts by constructing a regression model Y=f(X), where X represents molecular descriptors derived from molecular structures, and Y represents properties or activities. Next, a large number of chemical structures are generated computationally, which are then converted into X, and input into the regression model to predict the corresponding Y values. Molecules with favourable predicted property values can be selected. However, this approach restricts the search space to the set of candidate molecular structures, thereby limiting the comprehensiveness of the search. To overcome this limitation, several methods have been proposed to leverage generative models for the optimization of specific properties and activities. For instance, ACoVAE [18] defines inverse molecular design as a two‐step process involving the discovery of molecular descriptor vectors (seed vectors) expected to exhibit the desired activity, followed by the identification of corresponding chemical structures. A genetic algorithm (GA) and generative topographic mapping (GTM) are used to optimize the seed vectors, and VAE handles both the seed vectors and random latent vectors as inputs to generate molecules with the desired properties. Another approach uses GTM to explore the latent space of VAE [19]. This method identifies regions in the latent space enriched with molecules that possess properties of interest, and by sampling near these regions, it generates latent variables likely to correspond to active molecules. Furthermore, GENTRL [20] employs reinforcement learning within the VAE to efficiently train molecules with the target properties and activities, while DeepScaffold [21] generates molecules based on structural motifs possessing specific properties and activities. REINVENT [22] employs a reinforcement learning approach to finetune pre‐trained RNNs for generating molecules with given target properties.

**Figure 1:**
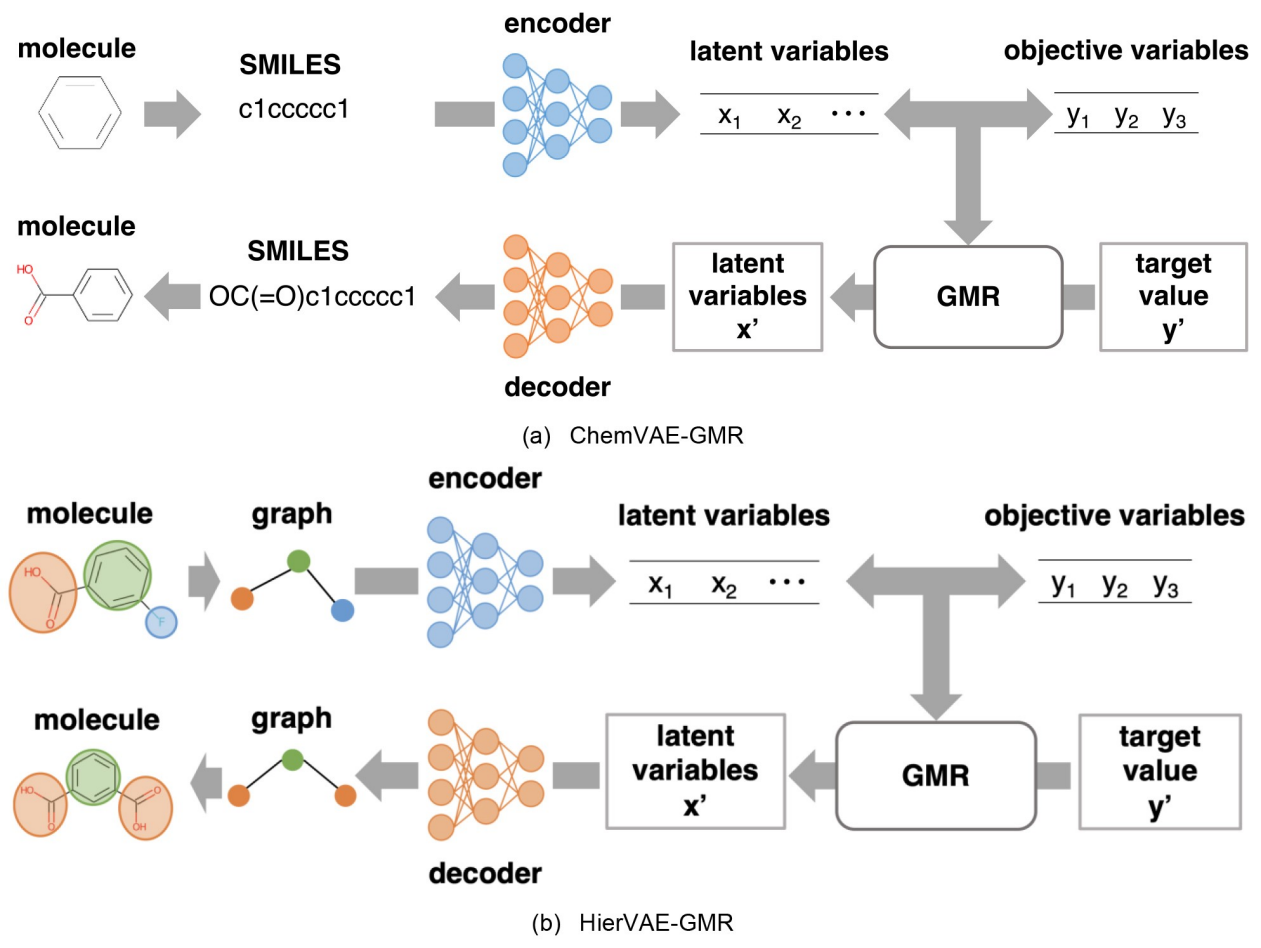
Basic concept of direct inverse analysis through the integration of VAE and GMR.

Additionally, a method has been proposed that allow for a more comprehensive exploration of chemical space by generating chemical structures directly from the desired values of properties Y. The present study investigates a method that combines ChemVAE [6] with Gaussian mixture regression (GMR) [23, 24] as a QSAR/QSPR model (ChemVAE‐GMR). ChemVAE‐GMR enables the direct generation of chemical structures from Y values, which can be applied to the optimization of properties and activities [25]. Figure 1a illustrates the basic concept of this method. First, the encoder and decoder are trained to match the input chemical structure with the output chemical structure using a large amount of molecular data. A mathematical model is then constructed between the latent variables X, derived from chemical structures via the encoder, and the target variable Y, which includes properties and activities. Through inverse analysis of this model, X values corresponding to desired target Y values are directly predicted. We can generate the chemical structure from these X values using the decoder and search for molecules with the desired Y values. In conventional techniques, chemical structures with a predicted Y that matches the target Y are selected from numerous virtual structures by pseudo‐inverse analysis. However, GMR allows chemical structures to be obtained directly from the target Y, enabling comprehensive searches of a vast molecular space.

Conventional molecule generation using VAEs sometimes produces invalid strings that do not conform to the SMILES grammar. Even in the case of valid SMILES, many unrealistic structures are generated, such as molecules with inappropriate bond orders or excessively large ring structures. Furthermore, the actual Y values of the generated molecules may be far from the target Y, making it challenging to generate molecules that simultaneously satisfy multiple target Y values.

In this study, we consider the task of generating realistic molecules and searching efficiently for molecules that simultaneously satisfy multiple target Y values. To achieve this, we focus on the hierarchical VAE (HierVAE) [11], a generative model using a graph structure, and combine this with GMR. ChemVAE treats SMILES as strings and approaches molecular generation as a string generation task [6]. However, the SMILES representation is not optimized for capturing molecular similarity. For instance, two molecules with similar chemical structures may be encoded into markedly different SMILES strings [10]. Therefore, ChemVAE struggles to accurately capture the grammar of SMILES, leading to the generation of ungrammatical strings and unrealistic structures. As a result, the generated molecules frequently do not achieve the target Y values. HierVAE addresses this problem through the use of a graph structure.

Although several benchmarking datasets such as MOSES [26] and GuacaMol [27], have been used to evaluate the performance of molecular generation, in this study, to fairly validate the effectiveness of the proposed method, we used the ZINC dataset [28] which were employed in previous studies [6, 7, 8, 10, 24] and use the water/octanol partition coefficient (logP), quantitative estimate of drug‐likeness (QED) [29], and synthetic accessibility score of drug‐like molecules (SAS) [30] as objective variables. We compare the results using our proposed HierVAE‐GMR against those from the conventional ChemVAE‐GMR. The data that support the findings of this study are available in ref [28].

## 2 Methods

### 2.1 HierVAE [11]

HierVAE is a graph structure‐based method. Initially, structural motifs are extracted based on substructures that frequently occur in the molecules of the training data. Molecules are represented as graphs, with motifs as nodes and bonds as edges. The decoder then constructs a molecule by combining large and small motifs. First, in the motif layer, the decoder predicts the next motif to be added based on the extracted motifs. Second, in the attachment layer, the decoder predicts candidate bonding sites at which the motifs can bond with each other and atoms in the intersection between neighbouring motifs. Finally, in the atom layer, the bonding of motifs is determined from the candidate bonding sites. By repeating the prediction in the above three layers, hierarchical molecular generation is achieved.

We have reference on HierVAE [11] for the architectural details of the model. In our model, we set the dimensions of both the hidden layers and embedding layers to 250, and the latent code dimension to 32. We also set the number of message passing iterations to be T=15. Python code from the web site given in ref [31] was used for the studies.

### 2.2 GMR [22, 23]

GMR is a regression analysis method that relies on the use of a Gaussian mixture model (GMM) to express a dataset through the superimposition of multiple Gaussian distributions. By combining X and Y and constructing a GMM, we obtain the joint probability distribution p(x, y) of X and Y. Estimating Y from X corresponds to obtaining the posterior distribution p(y|x) of Y given X. To compute p(y|x), we use the multiplication theorem and Bayes’ theorem applied to p(x, y). The distribution p(y|x) is represented by n normal distributions along with their respective weights. Finally, Y can be estimated in two ways: either by selecting the mean vector with the highest weight from the set of n normal distributions, or by calculating the weighted average of the n mean vectors. Although X and Y are explicitly treated separately, these variables are considered equivalent. Therefore, X can be estimated directly from Y by applying the same operations interchangeably with X and Y. This approach is known as direct inverse analysis. The method of DCEKit [32], a python toolkit, for GMR calculation.

### 2.3 HierVAE‐GMR

HierVAE‐GMR integrates the encoder and decoder of HierVAE with GMR. Figure 1b shows the basic concept of HierVAE‐GMR. In this approach, a molecule represented by a graph structure is transformed into latent variables by an encoder. Subsequently, a GMR model is constructed between these latent variables X and the target variable Y, which represents the desired properties and activities of the molecule. Once the target Y has been specified, X is obtained through direct inverse analysis of the GMR model. We then input X into the decoder to generate a molecule that has the specified target properties of Y.

### 2.4 Joint Training with Properties

**Figure 2:**
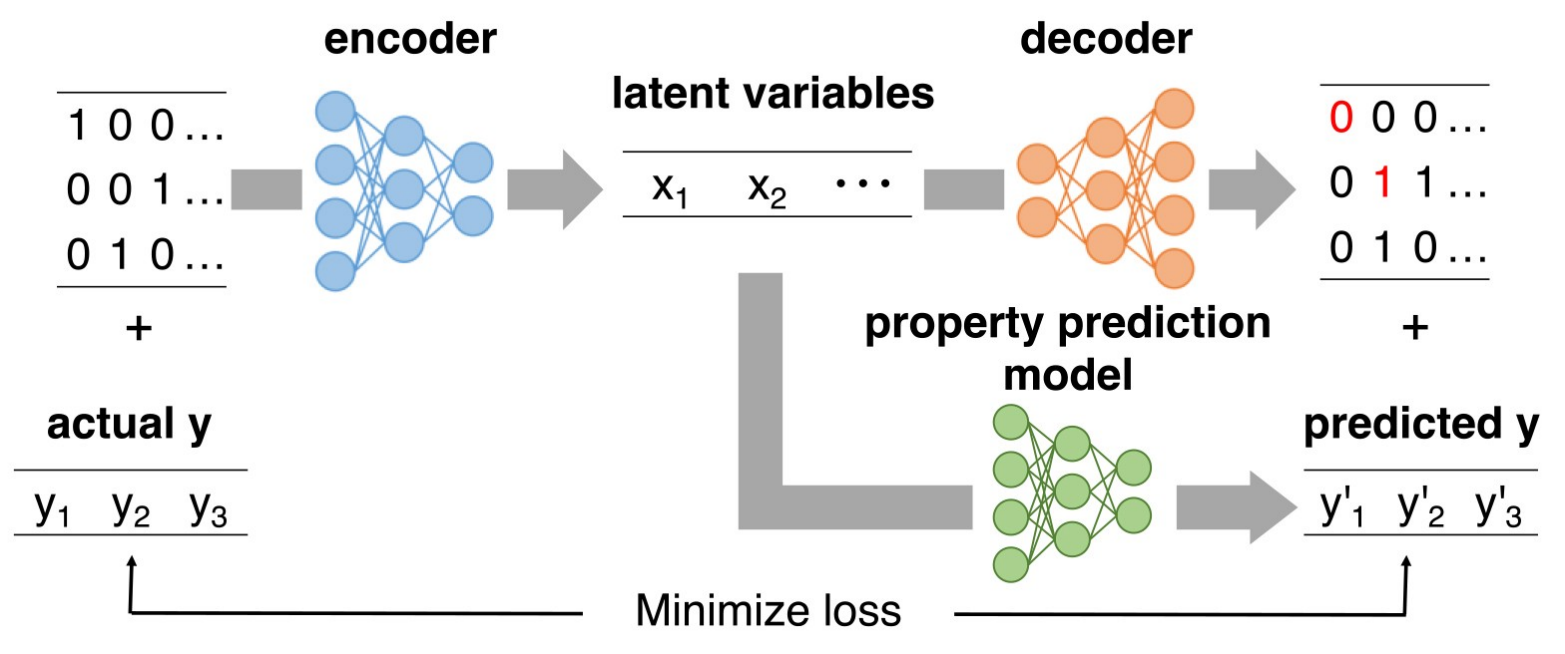
Diagram of VAE training with joint property prediction.

If the prediction accuracy of GMR is low, obtaining X that accurately reflects the target Y might be challenging, leading to the generation of inappropriate molecules. To overcome this issue, we jointly train the HierVAE on the property prediction task. Figure 2 illustrates the approach to joint training with properties. Conventional VAEs minimize both the reconstruction error between the input numerical data and the output data from the decoder, as well as the KL divergence between the latent space distribution and the prior distribution. In this study, we introduce a model that predicts properties from latent variables and calculate the loss between the predicted properties and the actual values. This loss is incorporated into the loss function of HierVAE and is designed to be minimized concurrently. This approach allows latent variables, which previously represented only chemical structure information, to represent the desired properties. This makes it possible to predict molecule properties using GMR.

The property prediction model employs a multilayer perceptron consisting of three layers with 67 neurons, trained with a dropout rate of 0.15. The target properties included logP, QED, and SAS, with their respective loss functions calculated using mean squared error (MSE). The loss function of the HierVAE adopted the negative ELBO, to which the MSE of the property tasks was added, and these are minimized simultaneously. Note that the CVAE does not use this loss function for training. We trained HierVAE with Adam optimizer with default parameters. The entire model construction and training process was performed using PyTorch [33] and RDKit [34].

## 3 Results and Discussion

We first used the proposed HierVAE‐GMR with joint training to predict molecule properties, i. e. logP, QED, and SAS. The logP value represents the logarithmically transformed octanol/water partition coefficient, which indicates the degree of oral absorption and significantly impacts drug development processes. QED serves as a quantitative measure of a chemical structure′s drug‐likeness, influenced by properties such as molecular weight, logP, and surface area polarity. A drug‐like molecule typically possesses a QED value close to 1, while a molecule lacking drug‐like properties tends to have a value close to 0. SAS is an index of synthetic accessibility reflecting the ease of synthesizing a molecule. A SAS value near 1 suggests easy synthesis, whereas a value near 10 indicates difficult synthesis. This metric considers factors such as molecular size, conformational size, and steric hindrance, and is derived from the analysis of extensive compound datasets and frequently occurring substructures. The properties were calculated by RDKit [34]. Latent variables served as explanatory variables. We used the ZINC dataset from ref [28] for our experiments. It contains about 250 K drug molecules extracted from the ZINC database [35]. For model training, we employed 10,000 randomly selected molecules from the dataset. We randomly selected 500 molecules not included in the training data for the test data. Cross‐validation and Bayesian optimization were used to optimize the hyperparameters.

**Figure 3:**
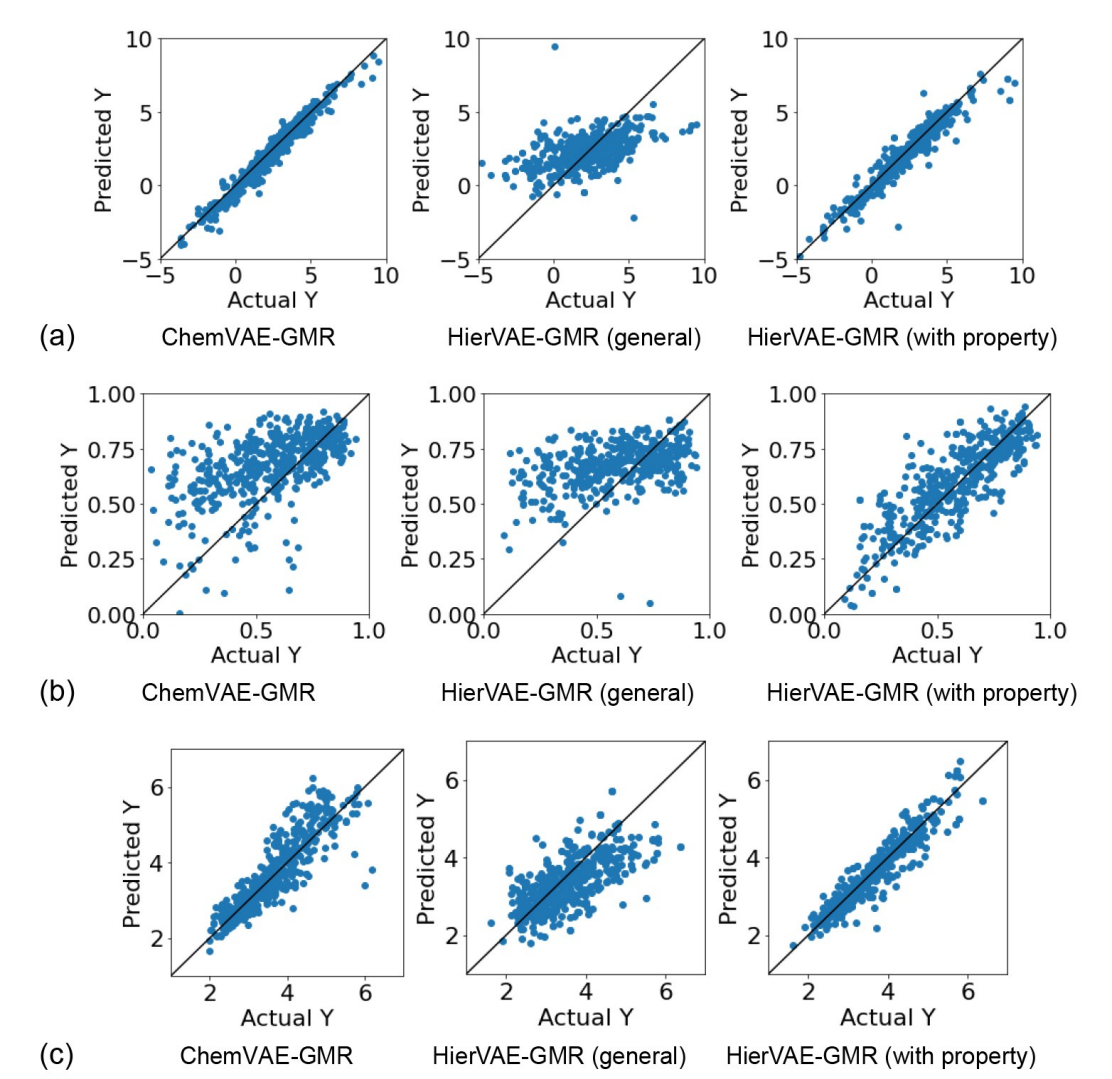
(a)Actual logP vs predicted value plots of test data. (b)Actual QED vs predicted value plots of test data. (c)Actual SAS vs predicted value plots of test data.

For validation, we compared the properties predicted using the conventional ChemVAE‐GMR with those given by HierVAE‐GMR (general) and HierVAE‐GMR (with property). The prediction results for each model are shown in Figure 3 and Table 1, where R^2^ is the coefficient of determination, RMSE is the root mean square error, and MAE is the mean absolute error. HierVAE‐GMR (general) achieves a lower prediction accuracy than ChemVAE‐GMR, whereas HierVAE‐GMR (with property) has a similar level of accuracy as ChemVAE‐GMR in terms of logP and significantly improves the prediction accuracy in terms of QED and SAS. The improvement is the result of jointly training the HierVAE with the molecular properties.

**Table 1:** Evaluation metrics for GMR model.

Property	Method	Evaluation metrics		
		R2	RMSE	MAE
logP	ChemVAE‐GMR	0.956	0.486	0.365
	HierVAE‐GMR (general)	0.215	2.021	1.505
	HierVAE‐GMR (with property)	0.911	0.682	0.415
QED	ChemVAE‐GMR	0.371	0.208	0.159
	HierVAE‐GMR (general)	0.274	0.214	0.166
	HierVAE‐GMR (with property)	0.783	0.117	0.091
SAS	ChemVAE‐GMR	0.805	0.455	0.321
	HierVAE‐GMR (general)	0.320	0.845	0.556
	HierVAE‐GMR (with property)	0.914	0.300	0.212

Next, molecular generation through direct inverse analysis was conducted using the proposed methods, and the results were compared with the baseline. The target values for inverse analysis were a logP of 2.00, a QED equal to the maximum value in the training data, and an SAS equal to the minimum value in the training data. Table 2 displays the number of outputs required to generate 1000 molecules and the number of these molecules meeting the threshold values. The threshold values were set to 1≤logP≤3, QED≥0.8, and SAS≤3.0. Furthermore, diversity and novelty of generated molecules were also evaluated in Table 2. The diversity metric^[11]^ is computed as the average pairwise molecular distance, which was the Tanimoto distance based on the Morgan fingerprints, between generated molecules which achieved the target values. The novelty metric was calculated as the proportion of generated molecules that are not present in the training data.

**Table 2:** Comparison of molecule generation results.

	Number of generation	Target molecules	Diversity	Novelty
ChemVAE‐GMR	225,200	0	–	–
HierVAE‐GMR (general)	1000	132	0.781	100%
HierVAE‐GMR (with property)	1000	169	0.794	100%

**Figure 4:**
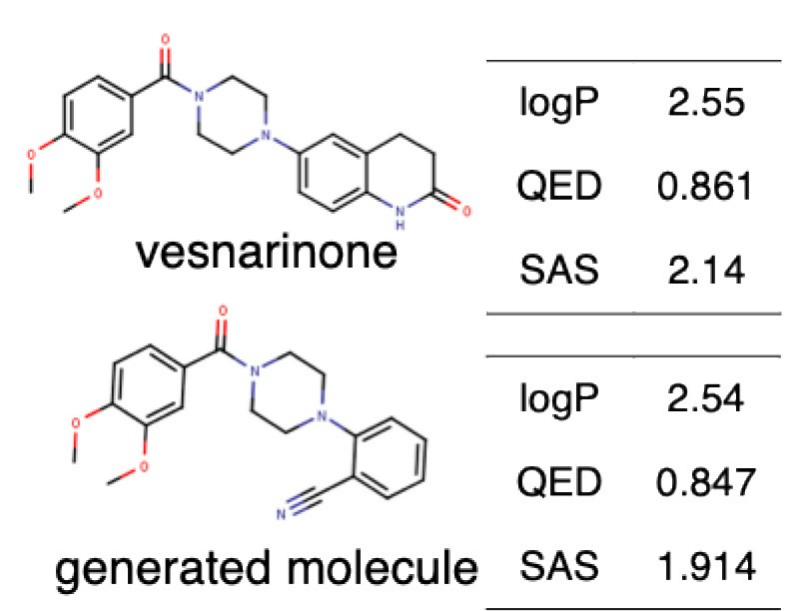
A molecule generated by the proposed method that achieve the target values.

ChemVAE‐GMR generates invalid molecules that do not satisfy the SMILES grammar, and required 225,200 outputs to generate 1000 molecules. In contrast, the proposed methods generated valid SMILES without issue and successfully generated 1000 molecules in 1000 outputs. None of the molecules generated by ChemVAE‐GMR reached the threshold values. The use of HierVAE‐GMR (general) generated 132 molecules satisfying the target values, whereas HierVAE‐GMR (with property) generated 169 molecules satisfying the thresholds. This highlights the significant superiority of our proposed method over the conventional one in terms of generating molecules with the desired properties. The diversity of the HierVAE‐GMR (with property) was slightly higher. This illustrates that, by training the HierVAE with the target properties, our property‐trained model can generate a greater number of molecules that satisfy the desired properties while maintaining molecular diversity. Figure 4 shows examples of the molecules generated by the HierVAE‐GMR (with property). MarvinView software in Chemaxon [36] was used to visualize the chemical structures. These molecules are more realistic than those given by ChemVAE‐GMR, with no unrealistic structures (i. e. large ring structures). Moreover, some of the molecules generated by the proposed method exhibit structures similar to drugs not included in the dataset. This suggests that our proposed method can produce drug‐like molecules with properties resembling those of real drugs.

**Figure 5:**
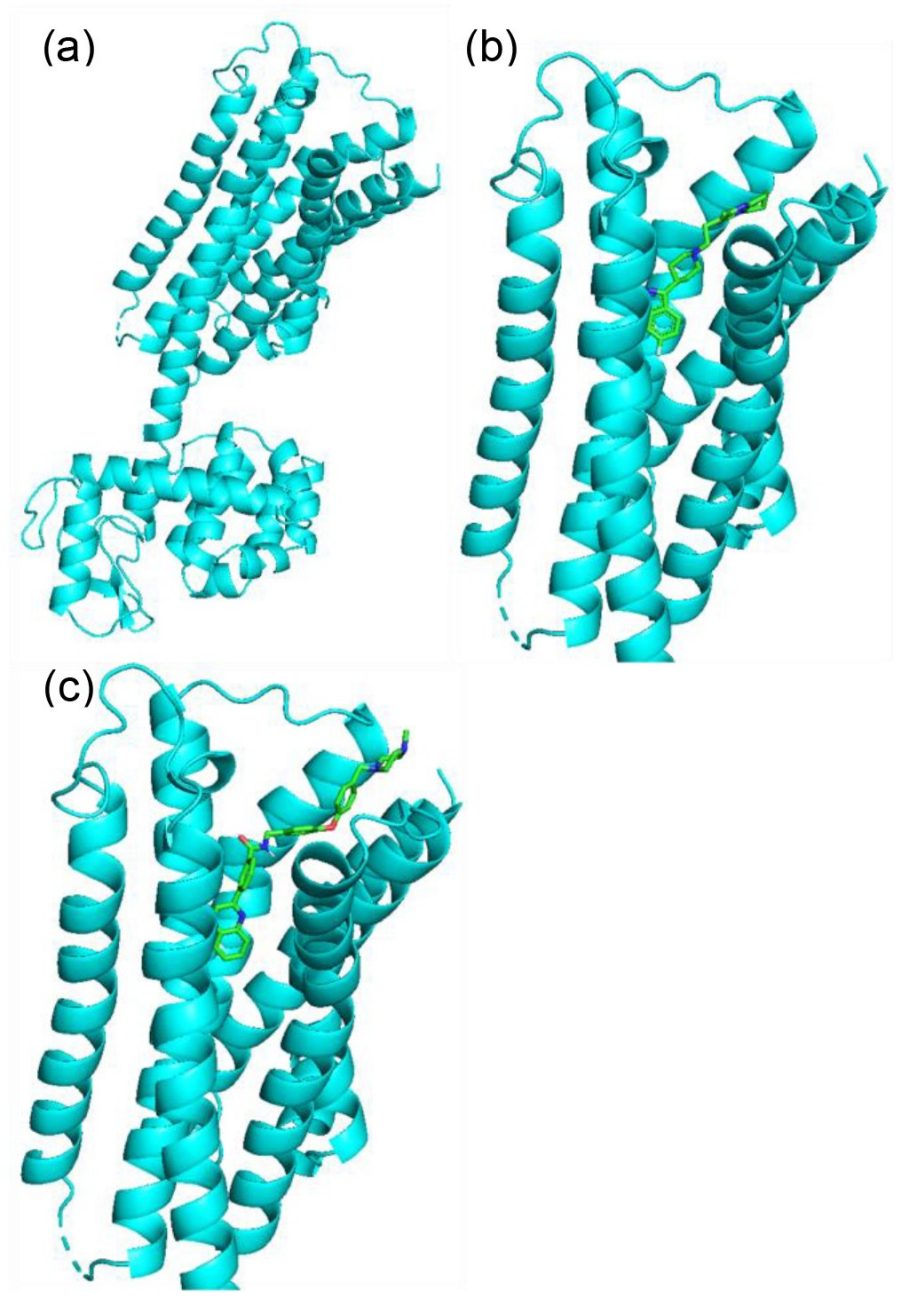
(a) Crystal structure of target protein (DRD2). (b) Docking pose of interaction between DRD2 and risperidone. (c) Docking pose of interaction between DRD2 and the novel molecule with the highest binding affinity.

Finally, we assessed the effectiveness of our proposed method on established drug discovery targets. D2 dopamine receptor (DRD2) is a well‐known target protein of antipsychotic drugs [37]. By performing protein‐ligand docking analysis for the generated molecules with the target proteins that interact with the antipsychotic drugs, we searched for novel molecules that are expected to have greater efficacy as molecular‐targeted drugs. Protein‐ligand docking analysis was conducted using AutoDock Vina (version 1.1.2) [38, 39]. The complex structure of DRD2 and Risperidone (PDB ID: 6CM4) [40] was used, from which Risperidone, water, and other solvents were removed from the PDB file. The input files for the protein structure were prepared using AutoDockTools [41]. Then, 3D conformers were generated from molecular structures, including Risperidone, using Chem.AllChem.EmbedMolecule() function of the RDKit by the ETKDG method [34]. Subsequently, these were converted into ligand input files using AutoDockTools. The docking site was set to cover the site where risperidone was bound in 6CM4. Figure 5a shows the crystal structure of DRD2, while Figure 5b shows the docking pose of interaction between DRD2 and risperidone. Docking was performed for each ligand, and the most stable docking pose along with its corresponding binding affinity energy (kcal/mol) was calculated. This analysis was integrated with the molecular generation process of HierVAE‐GMR (with property) to search for molecules with lower energies.

**Figure 6:**
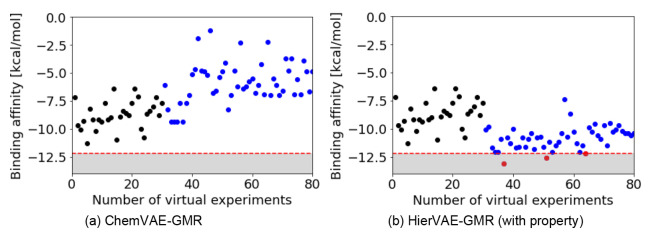
Binding affinity energies of generated molecules; black points indicate initial samples, blue points indicate generated samples, red points indicate samples meeting the target, grey area indicates target range (target value: −12.2).

Initially, we randomly selected 30 samples from the ZINC dataset and computed their energies. The GMR model was then trained using these initial samples. Subsequently, one sample was generated by direct inverse analysis and energy calculations, and this was incorporated into the training dataset. This process was repeated 50 times as a single cycle. The goal of this experiment was to achieve an energy lower than that of the original molecule (−12.2 kcal/mol). For comparison, we also validated ChemVAE‐GMR using the same procedure. The results are shown in Figure 6. Using ChemVAE‐GMR, no molecules with an energy lower than that of the existing ligand were generated after 50 cycles. In contrast, using HierVAE‐GMR (with property), the property of the ligand was gradually enhanced by iteratively generating molecules from the initial samples. Eventually, after the seventh generation, a molecule meeting the target was generated (see red dots in Figure 6). After 50 trials, we had successfully generated three molecules meeting the target properties, as detailed in Table 3. Figure 5c shows the docking pose of interaction between DRD2 and the novel molecule with the highest binding affinity. This demonstrates that our proposed method is effective in identifying molecules for drug targets.

**Table 3:** The original molecule and the molecules generated by the proposed method that exhibit the target properties.

Molecular structure	Cycle	Binding affinity [kcal/mol]
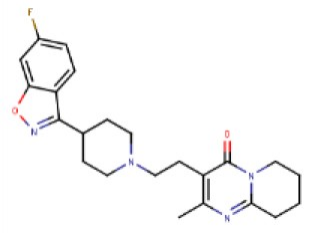		
risperidone: Cc1nc2n(c(=O)c1CCN1CCC(c3no c4cc(F)ccc34)CC1)CCCC2	–	−12.2
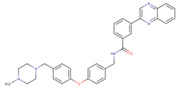		
CN1CCN(Cc2ccc(Oc3ccc(CNC(= O)c4cccc(c5cnc6ccccc6n5)c4)cc 3)cc2)CC1	7	−13.1
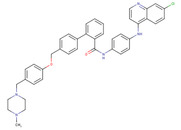		
CN1CCN(Cc2ccc(OCc3ccc(−c4 ccccc4 C(=O)Nc4ccc(Nc5ccnc6 cc(Cl)ccc56)cc4)cc3)cc2)CC1	21	−12.6
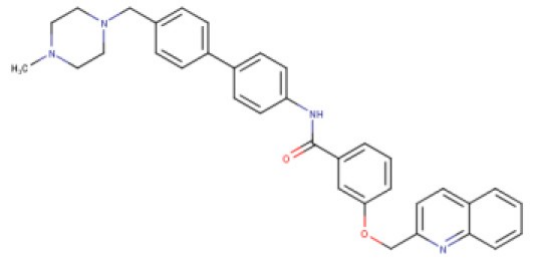		
CN1CCN(Cc2ccc(−c3ccc(NC(=O )c4cccc(OCc5ccc6ccccc6n5)c4) cc3)cc2)CC1	34	−12.2

## 4 Conclusions

To generate realistic molecules and efficiently search for molecules that simultaneously satisfy multiple targets, we developed a novel molecular design method. This method combines HierVAE, a graph‐structured VAE‐based model for molecular generation, with GMR to enable direct inverse analysis. Through joint training with molecule properties, our proposed method achieves significantly improved prediction accuracy of the target properties and activities. Notably, it enables the generation of chemical structures directly from the target values of objective variables and the design of chemical structures simultaneously satisfying multiple target values, which is challenging for conventional methods. Moreover, we identified several molecules that are predicted to exhibit activity against drug targets. As demonstrated in our docking results, even with limited data, the construction of the predictive GMR model could be achieved and molecules that effectively meet the target properties could be generated. These indicate that our proposed method will work even when the availability of labeled data is limited. While this study did not include fine‐tuning of the HierVAE model using docking scores, we believe that such fine‐tuning could enable the generation of molecules more closely aligned with this specific property. As a future work, we attempt this fine‐tuning approach to enhance the generation of molecules tailored to docking scores. Overall, this method is effective in searching for highly functional molecules in drug discovery and is expected to play an important role in the drug development process.

## Conflict of Interests

The author declares no conflicts of interest.

5

## Data Availability

The data that support the findings of this study are available in ref [28].
